# Roles of TRP and PIEZO receptors in autoimmune diseases

**DOI:** 10.1017/erm.2023.23

**Published:** 2024-04-25

**Authors:** Baoqi Yang, Dan Ma, Xueqing Zhu, Zewen Wu, Qi An, Jingwen Zhao, Xinnan Gao, Liyun Zhang

**Affiliations:** Third Hospital of Shanxi Medical University, Shanxi Bethune Hospital, Shanxi Academy of Medical Sciences, Tongji Shanxi Hospital, Taiyuan 030032, China

**Keywords:** Diabetes, inflammatory bowel disease, multiple sclerosis and gout, osteoarthritis, PIEZO, rheumatoid arthritis, Sjögren's syndrome, systemic lupus erythematosus, TRP

## Abstract

Autoimmune diseases are pathological autoimmune reactions in the body caused by various factors, which can lead to tissue damage and organ dysfunction. They can be divided into organ-specific and systemic autoimmune diseases. These diseases usually involve various body systems, including the blood, muscles, bones, joints and soft tissues. The transient receptor potential (TRP) and PIEZO receptors, which resulted in David Julius and Ardem Patapoutian winning the Nobel Prize in Physiology or Medicine in 2021, attracted people's attention. Most current studies on TRP and PIEZO receptors in autoimmune diseases have been carried out on animal model, only few clinical studies have been conducted. Therefore, this study aimed to review existing studies on TRP and PIEZO to understand the roles of these receptors in autoimmune diseases, which may help elucidate novel treatment strategies.

## Introduction

Autoimmune diseases refer to clinical disorders caused by internal and external factors, such as genetic and environmental factors. These lead to the destruction of autoimmune tolerance or abnormal regulation of autoimmune cells, and the continuous response of the immune system to self-antigens, resulting in damage or dysfunction. Autoimmune diseases are divided into organ-specific and systemic autoimmune diseases. Organ-specific autoimmune diseases include Hashimoto's thyroiditis, exophthalmic goitre and insulin-dependent diabetes mellitus. Systemic autoimmune diseases include rheumatoid arthritis (RA), osteoarthritis (OA), systemic lupus erythematosus (SLE), and Sjögren's syndrome (SS). The 2021 Nobel Prize in Physiology or Medicine drew people's attention to the transient receptor potential (TRP) ion channels and PIEZO receptors. Both TRP and PIEZO have been studied in autoimmune diseases. Therefore, this study aimed to review the most recent literature on TRP and PIEZO in autoimmune diseases.

## TRP and autoimmune diseases

TRP ion channels are a class of channel proteins widely distributed in the peripheral and central nervous systems. To date, more than 30 members of the TRP channel family have been cloned in mammals. TRP channels are six-transmembrane proteins with intracellular N-termini and C-termini. Additionally, the fifth and sixth transmembrane domains together constitute a non-selective cation channel. The TRP channel family includes seven subfamilies, divided into two categories. The first category includes TRPC (typical), TRPV (capsaicin), TRPM (M-type), TRPN (no mechanoreceptor potential) and TRPA (ankyrin). The second category includes TRPP (polycystin) and TRPML (mucolipoprotein). Six TRP channel subfamilies (excluding TRPN) have been confirmed in mammals. These channels can be regulated by many factors, including temperature, osmotic pressure, pH, mechanical force, endogenous and exogenous ligands and intracellular signalling molecules. Currently, the most recognized function is to mediate the transmission of sensory signals, including temperature, pain, pressure, vision and taste, and to regulate cellular calcium balance and affect development. TRP channels are widely expressed in the sensory neurons, skin and brain. Furthermore, studies have shown that TRPV1 and TRPA1 can be endogenously expressed in T cells and participate in T-cell activation and cytokine secretion (Refs 1, 2). TRP channels in the free nerve terminals of sensory neurons convert painful thermal (TRPV1, TRPA1, TRPM8 and TRPM3), chemical (TRPA1) and mechanical (TRPC1, TRPC3, TRPC6, TRPA1, TRPV2 and TRPV4) stimuli into electrophysiological stimuli. Some TRP channels are not involved in physiological sensation but do play a role in pathological conditions. For example, TRPC5 in mechanical sensation, and TRPV1 and TRPA1 in keratinocytes and satellite glia are involved in the transduction of pain stimuli. In addition, TRP mainly senses temperature changes. Imbalances in temperature perception can shift the comfort temperature from innocuous to noxious stimuli, resulting in pathological pain conditions. Since the gating mechanism of TRP is also regulated by certain inflammatory mediators, these channels are thought to be involved in other pathological pain states, such as inflammatory hyperalgesia and diabetic neuropathy (Ref. 3).

Most autoimmune diseases present with symptoms of pain. Opioids are currently used in pain treatment but have addictive side effects and are prone to abuse. In contrast, commonly used painkillers for autoimmune diseases, such as non-steroidal anti-inflammatory drugs, are associated with the risk of cardiovascular diseases and gastrointestinal problems. Therefore, there is an urgent need for safe and effective painkillers. Since TRP is widely expressed in sensory neurons, skin and brain, targeting TRP may represent a novel therapeutic approach for pain treatment. TRP receptors have also been studied in autoimmune diseases. The following is a review of research on TRP in common autoimmune diseases.

### Organ-specific autoimmune diseases

#### Diabetes

Diabetes is a group of metabolic diseases characterized by hyperglycaemia. Hyperglycaemia is caused by defective insulin secretion, impaired biological action or both. Long-term hyperglycaemia leads to chronic damage and dysfunction of various tissues, particularly the eyes, kidneys, heart, blood vessels and nerves. Diabetes can be divided into type 1 diabetes mellitus (T1DM) and type 2 diabetes mellitus (T2DM). T1DM develops at a young age (usually <30 years old), with sudden onset and obvious symptoms of polydipsia, polyuria, polyphagia, weight loss and high blood sugar level. Many patients also experience ketoacidosis as the first symptom and demonstrate low serum insulin, C-peptide levels, islet cell antibodies (ICA), insulin autoantibodies (IAA) and glutamate decarboxylase antibodies (GAD-Ab). Oral medication alone is ineffective, and insulin therapy is required. Additionally, T2DM is common in middle-aged and older individuals, and the incidence of obesity is high in these individuals. T2DM is often accompanied by hypertension, dyslipidaemia, arteriosclerosis and other diseases, with an insidious onset, asymptomatic early stages or only mild fatigue, thirst and no obvious increase in blood sugar levels. A glucose tolerance test can be performed for diagnosis. Serum insulin levels are normal or increased in the early stages and decreased in the late stage.

*T1DM.* Animal models have shown that TRPC1 gene expression is significantly reduced during the late stages of diabetic nephropathy (DN), however clinical studies demonstrated that TRPC1 gene polymorphism may not fundamentally contribute to the development of DN (Refs 4, 5).

Additionally, TRPC6 expression was increased in DM rats and mice, and TRPC6 gene knockout was associated with decreased albuminuria in young animals (12-16 weeks of age) in a T1DM mouse model. However, this protective effect was no longer observed once the animals reached 20 weeks of age. A combined knockout of TRPC3, TRPC6, and TRPC7 reduced albuminuria and histological kidney injury. In the rat model, TRPC6 knockout had no significant protective effect against hyperglycaemia, albuminuria, histological kidney injury, blood urea nitrogen, or serum creatinine (Refs 6, 7).

In a spontaneous T1DM model, non-obese diabetic (NOD) mice were subcutaneously injected with high-dose capsaicin, which can inactivate TRPV1 sensory nerve endings (Ref. 8), significantly reduced islet inflammation, delayed the onset of diabetes, and had no effect on the autoimmune invasion of other tissues. In other words, in the absence of TRPV1-positive neurons in the pancreas, protection against diabetes was not due to the elimination or dysregulation of the diabetic T cell population. Capsaicin-treated NOD mice still possess similar numbers of islet-reactive T cells compared with the NOD control mice (Ref. 9). Individuals with T1DM have a lower bone mineral density and a higher risk of fractures. The role of osteoblasts in diabetes-related osteoporosis is well established; however the role of osteoclasts (OCL) is unclear. Furthermore, in vitro studies have shown that diabetes causes local acidosis in the bone marrow, which stimulates OCL formation by activating TRPV1 (Ref. 10).

Compared with healthy controls, TRPM7 expression was increased in the hippocampus of T1DM mice. TRPM7 gene silencing can significantly inhibit the decrease of body weight and fasting insulin; inhibit the increase of blood glucose, ICA, IAA, and GAD-Ab levels in streptozotocin-induced; improve spatial cognitive function; and protect hippocampal neurogenesis in T1DM mice (Ref. 11).

**Figure 1.**
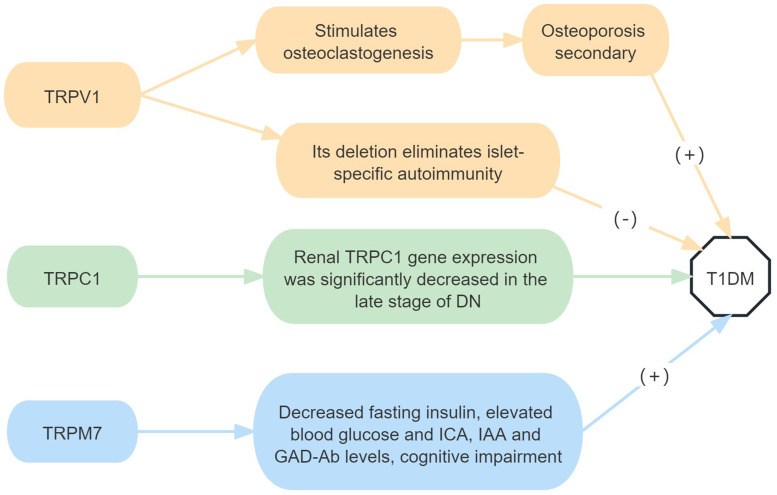
Roles of TRP receptor subtypes in T1DM. *Note*: (+) means to promote the onset of disease, and (−) means to inhibit the onset of disease. DN, diabetic nephropathy; ICA, islet cell antibodies; IAA, insulin autoantibodies; GAD-Ab, glutamate decarboxylase antibodies.

In conclusion, TRPV1 and TRPM7 activation promotes T1DM. At the same time, TRPV1 can also stimulate OCL formation, leading to osteoporosis secondary to T1DM, and TRPM7 activation is also related to cognitive dysfunction in T1DM (Fig. 1).

*T2DM.* The TRPC1 single nucleotide polymorphisms (SNPs) rs7638459 and rs953239 were susceptibility loci associated with T2DM and T2DM without DN, respectively. Among the rs7638459 polymorphisms, the CC genotype significantly increased the risk of T2DM compared with the TT genotype. rs953239 was significantly associated with DN in T2DM, and the CC genotype significantly reduces the risk of DN compared with the AA genotype (Ref. 12).

TRPC6 is expressed in podocytes and mediates podocyte injury induced by high glucose levels, a study shows that tacrolimus protects podocytes during the progression of type 2 DN, possibly ameliorating podocyte apoptosis by downregulating the expression of TRPC6 (Ref. 13). TRPC6 knockout may reduce the intracellular calcium overload in the brains of T2DM mice, thereby reducing amyloid beta protein deposition and neuroinflammation, and ultimately delaying the development of T2DM-related cognitive dysfunction (Ref. 14).

In rodent models of T2DM, pharmacological blockade of TRPV1 by small-molecule antagonists inhibited calcitonin gene-related peptide (CGRP) secretion, stimulated insulin secretion, reduced insulin resistance and prevented disease progression (Refs 15, 16, 17). However, it was shown that the TRPV1 agonist capsaicin reduces obesity-induced inflammation, insulin resistance and hepatic steatosis in obese mice fed a high-fat diet (HFD), and reduces fasting blood glucose levels (Refs 18, 19). Simultaneous TRPV1 activation also enhances intestinal glucagon-like peptide-1 (GLP-1) secretion and improves glucose homeostasis (Ref. 20).

In rodent models of T2DM, TRPV4 agonists promote vasodilation and improve cardiovascular function, whereas TRPV4 antagonists reduce HFD-induced obesity, insulin resistance, DN, retinopathy and neuropathy (Ref. 21).

TRPM2 silencing significantly reduces fibrosis and inflammation in the kidneys of HFD-induced diabetic mice, largely by inhibiting transforming growth factor-*β*1 (TGF-*β*1) activation (Ref. 22).

TRPM5 is a non-selective monovalent cation channel expressed in islet *β* cells and activated by an increase in intracellular calcium ions (Ca^2+^). Islets in TRPM5-knockout mice have shown a significant reduction in glucose-induced calcium activity insulin release, insulin secretion and glucose clearance in both intraperitoneal glucose tolerance tests and oral glucose tolerance tests (Ref. 23). In addition to glucose-induced insulin secretion, L-arginine-induced insulin secretion (in the presence of low glucose levels) is also impaired in TRPM5-knockout mice, but this effect can be suppressed by the TRPM5 inhibitor triphenylphosphine oxide (TPPO). Furthermore, TPPO blockade of TRPM5 inhibits GLP-1-enhanced glucose-induced insulin release (Refs 24, 25, 26). These findings suggest that TRPM5 expression levels inversely correlate with T2DM development (Ref. 27).

There is no evidence of an association between common TRPM6 and TRPM7 haplotypes and diabetes risk. Compared with non-carriers, TRPM61393Ile-1584Glu haplotype carriers have an increased risk of T2DM only with low magnesium intake (<250 mg/day) (Ref. 28).

Moreover, allyl isothiocyanate, a potent TRPA1 agonist, may have beneficial effects on glucose uptake and amelioration of impaired insulin signalling through TRPA1 activation. The protective effect against insulin resistance has also been associated with increased mitochondrial activity (Ref. 18).

**Figure 2.**
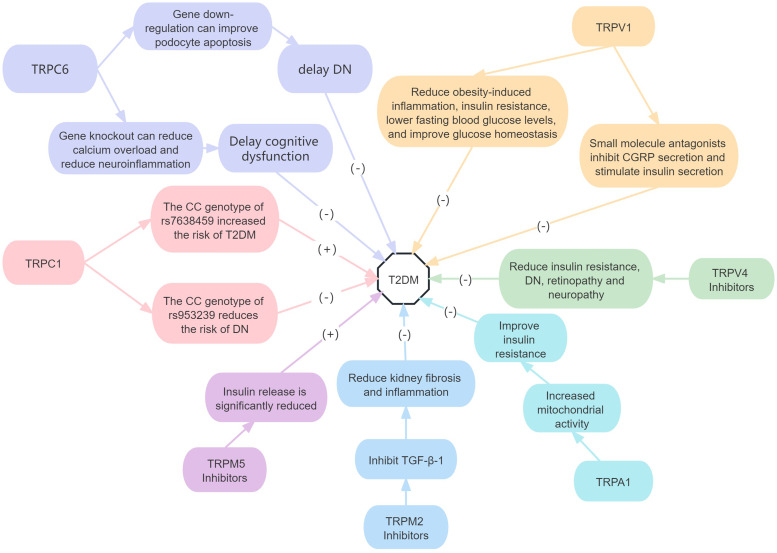
Roles of TRP receptor subtypes in T2DM. *Note*: (+) means to promote the onset of disease, and (−) means to inhibit the onset of disease. CGRP, calcitonin gene-related peptide; TGF-*β*1, transforming growth factor-*β*1.

In conclusion, TRPC1 SNPs are associated with an increased risk of developing T2DM and DN in T2DM. TRPM5 and TRPA1 activation can improve insulin secretion and negatively correlate with T2DM development, whereas TRPC6, TRPV4 and TRPM2 antagonism can slow down diabetes progression. However, studies on TRPV1 in T2DM have contradictory results; therefore, further research is needed. Studies on the other TRP subfamilies in T2DM are also lacking (Fig. 2).

#### Inflammatory bowel disease (IBD)

IBD is a group of idiopathic intestinal inflammatory diseases that involve the ileum, rectum and colon. Clinical manifestations include diarrhoea, abdominal pain and bloody stools. This group includes ulcerative colitis (UC) and Crohn's disease (CD). UC is characterized by continuous inflammation of the colonic mucosa and submucosa, usually first involving the rectum with gradually spreading to the entire colon. CD can involve the entire digestive tract and is a discontinuous full-thickness inflammation. The most involved sites are the terminal ileum, colon and anus.

Intestinal fibrosis is a refractory complication of CD in which TGF-*β*1 significantly upregulates TRPC6 mRNA and protein expression in patients with CD. Upregulation of TRPC6 expression is critical to the formation of *α*-smooth muscle actin stress fibres and N-cadherin-mediated adhesion junctions, which enable myofibroblasts to acquire contractile force and strengthen the interconnection between cells simultaneously. Increased Ca^2+^ influx is involved in the development of intestinal fibrosis by negatively regulating the synthesis of anti-fibrotic factors (Refs 29, 30).

Studies have shown that TRPV1 has a pro-inflammatory effect, and inflammatory mediators activate TRPV1 receptors and induce neurogenic inflammatory components through the release of substance P (SP), neurotensin, vasoactive intestinal polypeptide and galanin. In particular, TRPV1 gene-deficient mice exhibit less dextran sodium sulphate (DSS)-induced colitis (Refs 31, 32). Intraperitoneal and intrathecal administration of TRPV1 and TRPA1 antagonists exerts analgesic effects in a rat colitis model that emphasizes central nervous system mechanisms (Ref. 33). However, Massa *et al*. showed that more severe dinitrobenzenesulfonate (DNBS)-induced colitis was observed in TRPV1-knockout mice, suggesting a protective effect of TRPV1 (Ref. 34). In addition, oral curcumin alleviates visceral hyperalgesia by inhibiting TRPV1 phosphorylation in a rat model of UC (Ref. 35). In a clinical trial, TRPV1 nerve fibres were increased in quiescent IBD with irritable bowel syndrome-like symptoms and correlated with pain severity. Further studies have shown that TRPV1 expression is increased in inflammatory tissues of patients with active UC compared with non-inflamed tissues and is associated with disease recurrence and persistent activity (Refs 36, 37). Another clinical study showed that TRPV1 expression was significantly upregulated in the colonic epithelium of patients with IBD compared with controls, and TRPV1 expression was not significantly different between groups with UC and CD. Although TRPV1 was highly expressed in epithelial cells and infiltrating inflammatory cells in colon biopsies from patients with active IBD, TRPV1 expression was not significantly associated with disease severity (Ref. 38). Activation of TRPV1 receptors on sensory nerve terminals mediates neurogenic inflammation through the release of SP and CGRP, resulting in increased vascular permeability, plasma protein extravasation and inflammatory cell activation. While anti-inflammatory sensory neuropeptides, such as somatostatin and opioid peptides, released simultaneously from the same nerve terminal, exert anti-inflammatory and analgesic effects locally and systemically by entering the circulation. Therefore, the role of TRPV1 in IBD depends on a variety of neurotransmitters and cytokine interactions (Ref. 39).

TRPV4 activation triggers pro-inflammatory signals in the gut. After TRPV4 agonists are injected into the colon cavity of mice, the expression of pro-inflammatory chemokines and cytokines upregulates in mouse tissues, myeloperoxidase activity significantly increases and inflammatory cells infiltrate. Inhibition of TRPV4 activation is protective in a colitis model (Refs 40, 41, 42).

TRPM2 may play a pro-inflammatory role in colitis through its essential roles in macrophages and nuclear factor kappa-B (NF-*κ*B) signalling. Immune cell infiltration and intestinal inflammation severity have been improved in TRPM2-knockout mice with DSS-induced colitis (Ref. 43).

In animal experiments, TRPM8 exerted a protective effect in the intestine by inhibiting the release of tumour necrosis factor alpha (TNF-*α*), interleukin (IL)-1, IL-6, monocyte chemoattractant protein-1 and CGRP. TRPM8 activation also reduces TRPV1-dependent CGRP release in the gut; therefore, TRPM8 is capable of suppressing TRPV1-related inflammatory cascades (Refs 44, 45).

In an animal study, mice with experimental colitis exhibited increased TRPA1-mediated release of colonic neuropeptides, whereas symptoms were reduced after TRPA1 inhibition by antagonists or gene deletions (Ref. 46). Other studies have shown that TRPA1 plays a protective role in the gastrointestinal tract, and carnabiverine, a potent TRPA1 agonist, improves DNBS- and DSS-induced neutrophil infiltration, intestinal permeability, and cytokine, neuropeptide and chemokine production, and alters the dysbiosis of the gut microbiota (Refs 47, 48). Compared with the control group, TRPA1-knockout mice showed more significant intestinal fibrosis, potentially related to the anti-fibrotic effect of TRPA1 in intestinal myofibroblasts (Refs 49, 50). A clinical study showed that carnabiverine reduced intestinal inflammation in children with active UC (Ref. 47). TRPA1 promoter methylation is dysregulated in patients with CD, and abnormal TRPA1 expression may lead to typical symptoms of CD (Ref. 51). Therefore, TRPA1 may play a protective or destructive role in colitis.

**Figure 3.**
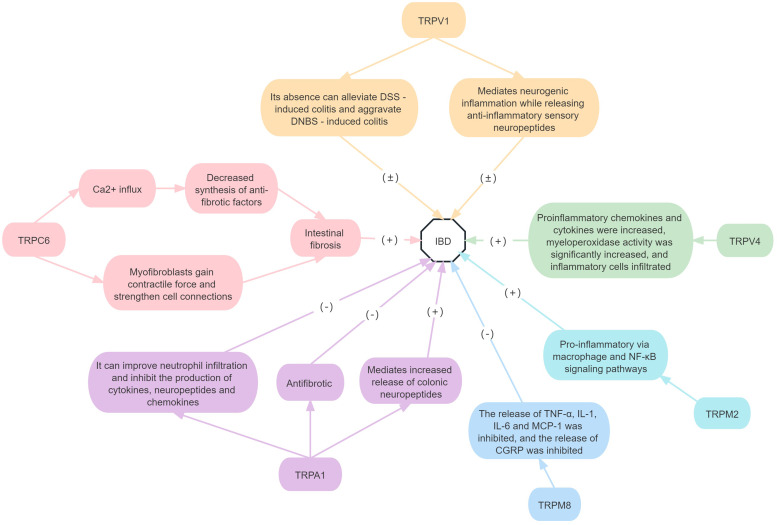
Roles of TRP receptor subtypes in IBD. *Note*: (+) means to promote the onset of disease, and (−) means to inhibit the onset of disease. DSS, dextran sodium sulfate; DNBS, dinitrobenzenesulfonate; CGRP, calcitonin gene-related peptide; NF-*κ*B, nuclear factor kappa-B; TNF-*α*, tumour necrosis factor alpha; IL, interleukin; MCP-1, monocyte chemoattractant protein-1.

In conclusion, TRPC6 promotes the development of intestinal fibrosis. Additionally, TRPV4 and TRPM2 play a pro-inflammatory role in IBD, while TRPM8 inhibits the inflammatory response. The research results of TRPV1 and TRPA1 roles in IBD are, however, inconsistent; they may promote or inhibit disease progression. There are no relevant studies on the roles of the remaining TRP subfamilies in IBD (Fig. 3).

#### Multiple sclerosis (MS)

MS is the most common demyelinating disease of the central nervous system. It is characterized by acute active central nerve white matter with multiple inflammatory demyelinating spots and old lesions of calcified spots due to glial fibre hyperplasia. The disease is also marked by multiple lesions, remission and recurrence. It is more likely to occur in the optic nerve, spinal cord and brain stem and is more common in young and middle-aged women than in men.

TRPV1 channels regulate ATP-induced NLR family pyrin domain containing 3 (NLRP3) inflammasome activation by regulating calcium influx and protein phosphatase 2A phosphorylation in microglia. Furthermore, TRPV1 deletion attenuates experimental autoimmune encephalomyelitis (EAE) and neuroinflammation in mice by inhibiting NLRP3 inflammasome activation (Ref. 52). TRPV1 may play a role in pain and weakness associated with influenza-like syndrome in INF*β*-induced relapsing-remitting MS (RRMS), especially in GG carriers of the TRPV1 SNP rs222747 (Ref. 53). In vitro, capsaicin stimulation of TRPV1 significantly reduced TNF and IL-6 release from activated microglia and in patients with MS. There was a significant correlation between the TRPV1 SNP rs222747 and cerebrospinal fluid (CSF) TNF levels; in particular, the presence of the G allele known to increase TRPV1 protein expression and function is associated with reduced CSF levels of TNF (Ref. 54). The findings suggest that TRPV2 may play a key role in myelination and could be an interesting clinical target for the treatment of demyelinating diseases (Ref. 55).

Genetic deletion of TRPM2 prevents dicyclohexanone oxalyl dihydrazone-induced demyelination, synapse loss, microglial activation, NLRP3 inflammasome activation and pro-inflammatory cytokine production, ultimately leading to improved cognitive decline (Ref. 56). In TRPM2-knockout mice, a reduced CXCL2 expression in the CNS inhibits neutrophil infiltration and slows down EAE progression (Ref. 57).

TRPM4 is expressed in mouse and human neuronal bodies, as well as in axons from inflammatory CNS injury in mouse EAE and human MS tissue. The pharmacological inhibition of TRPM4 by the antidiabetic drug glyburide resulted in reduced axonal and neuronal degeneration and lower clinical disease scores in EAE, but this did not alter EAE-related immune function (Ref. 58).

TRPM7 overexpression in MS astrocytes inhibits axonal growth by regulating the production of chondroitin sulphate proteoglycans, a key component of gliosis scarring (Ref. 59). TRPM7 kinase mutation reduces cytokine production, including IL-17 and interferon gamma (IFN-*γ*), in mouse T cells, which in turn affects MS progression (Ref. 60).

TRPA1 is expressed in astrocytes in the central nervous system of mice. A previous study demonstrated that TRPA1 deficiency significantly attenuates dicyclohexanone oxalyl dihydrazone-induced demyelination by reducing apoptosis of mature oligodendrocytes (Ref. 61). TRPA1 is involved in the development of neuropathic pain, and pain of central nervous origin is the main symptom of spinal cord injury in RRMS. Another study showed that TRPA1 is associated with the development of mechanical and cold allodynia in a mouse model of relapsing–remitting EAE (RR-EAE)-induced central neuropathic pain (Refs 62, 63). Headaches are also frequent in patients with progressive MS (PMS). Enhanced TRPA1 endogenous agonists and NADPH oxidase activity have been detected in the trigeminal ganglia of PMS-EAE and RR-EAE mice, activating TRPA1 in trigeminal nociceptors, thereby inducing periorbital mechanical hyperalgesia (Refs 64, 65). The PMS-EAE model induces depressive and anxiety-like behaviours; however, a selective TRPA1 antagonist (A-967079) reverses these behaviours, suggesting that TRPA1 plays a fundamental role in depression and anxiety-like behaviours (Ref. 66). Lysophosphatidylcholine (LPC) is a key inducer of MS caused by neuronal inflammation and demyelinating syndrome. TRPA1 can mediate calcium overload, reactive oxygen species (ROS) generation, mitochondrial membrane depolarization, nitric oxide increase, superoxide production and cytotoxicity in OLN-93 oligodendrocytes induced by LPC, suggesting that TRPA1 plays an important role in LPC-induced oxidative stress and cell damage in OLN-93 oligodendrocytes. TRPA1 inhibition may protect against LPC-induced demyelination (Refs 67, 68).

**Figure 4.**
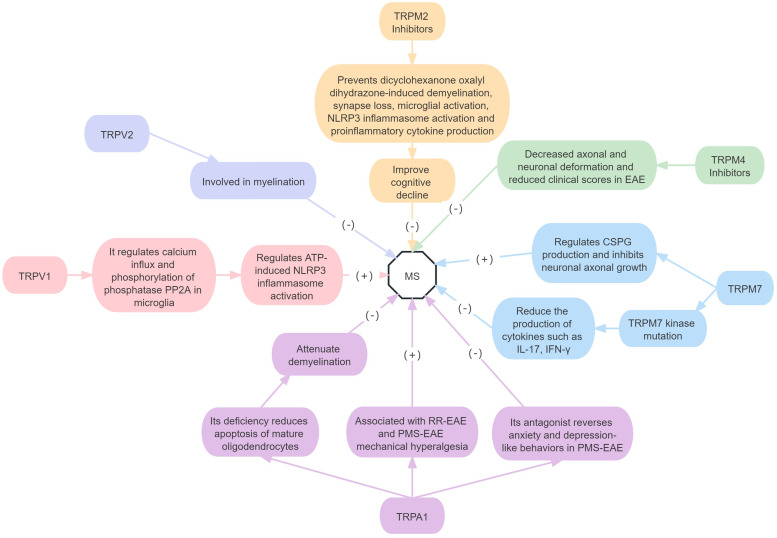
Roles of different TRP receptor subtypes in MS. *Note*: (+) means to promote the onset of disease, and (−) means to inhibit the onset of disease. EAE, experimental autoimmune encephalomyelitis; PMS, progressive multiple sclerosis; RR-EAE, relapsing-remitting experimental autoimmune encephalomyelitis; CSPG, chondroitin sulphate proteoglycans; IL, interleukin; IFN-*γ*, interferon gamma; PP2A, protein phosphatase 2A.

In summary, findings on the role of TRPV1 in MS are contradictory, and further research is needed. TRPV2 may be involved in myelination, TRPM2, TRPM4, TRPM7 and TRPA1 all play a harmful role in MS; there are no related studies on the roles of the other TRP subfamilies in MS (Fig. 4).

#### Anti-glomerular basement membrane autoimmune glomerulonephritis (GN)

In a rat model, the expression of TRPC6 in the glomerulus of rats treated with anti-glomerular basement membrane serum had significantly increased compared to rats treated with control serum. TRPC6 gene knockout could alleviate the pathological changes such as glomerular sclerosis; however, it does not reduce the related indexes such as total albuminuria, blood urea nitrogen and immunoglobulin (Refs 6, 69).

### Systemic autoimmune diseases

#### RA

RA is a chronic multifactorial autoimmune disease mainly characterized by inflammatory synovitis and involves multiple systems. It is also characterized by polyarticular, symmetrical and invasive inflammation of the small joints of the hands and feet, often accompanied by extra-articular organ involvement and positive serum rheumatoid factor, resulting in joint deformity and loss of function.

TRPC5 activation may be associated with the endogenous anti-inflammatory pathways that limit disease progression. In mice with complete Freund's adjuvant (CFA)-induced arthritis, inhibition of TRPC5 through gene deletion or pharmacological antagonism resulted in increased neutrophil infiltration; increased concentrations of cytokines such as IFN-*γ*, TNF-*α* and IL-10; and synovial angiogenesis, leading to increased active joint inflammation, hyperalgesia and synovitis. Compared with a non-arthritic control group, TRPC5 expression in patients with RA showed a decreasing trend, but this was not significant. TNF receptor and vascular adhesion molecule-1 expression was significantly increased in arthritic synovium (Ref. 70). Both mRNA and protein expression of TRPC6 were found somewhat higher levels in RA-FLSs than in OA-FLSs. Moreover, inhibiting expression of TRPC6 in vitro reduced proliferation of, as well as inflammatory mediator and protease production by, RA-FLSs, attenuates FLS-mediated synovial inflammation and joint destruction in RA (Ref. 71).

In a CFA-induced arthritis model, TRPV1 deletion reduced joint and paw swelling, synovial inflammation, bone erosion and cartilage damage in early stages (≤5 weeks) and suppressed RA-related pain later (>8 weeks) (Refs 72, 73, 74, 75). TNF-*α* plays an important role in RA with elevated levels in the synovial fluid of patients with RA (Ref. 76). TNF-*α*-mediated hyperalgesia is dependent on TRPV1 channels (Ref. 77). However, a previous study showed that SA13353 (1-[2-(1-adamantyl)ethyl]-1-pentyl-3-[3-(4-pyridyl)propyl]urea), an active TRPV1 agonist, inhibits TNF-*α* production by activating TRPV1-mediated capsaicin-sensitive afferent neurons, and reduces hind paw swelling and joint destruction in rats with collagen-induced arthritis (Ref. 78). TRPV1 activation in synovial fibroblasts (SF) from patients with RA lead to the production of the inflammatory molecules prostaglandin E2, IL-6 and IL-8, which mediate pain in inflamed joints (Refs 79, 80). Synthetic cannabinoids (WIN) below 1*μ*M play an anti-inflammatory role by reducing the production of cytokines such as IL-6, IL-8 and matrix metalloproteinase (MMP)-3 in RA SF through TRPV1- and TRPA1-dependent pathways (Ref. 81). Despite the increased functional expression of the TRPV2 gene in synovial cells from patients with RA, TRPV2 agonists reduced the in vitro invasiveness of these SF (Ref. 82). In a rat model of RA, the synovial fluid in the joint is abnormal, accompanied by decreased tension, increased acidity and accumulation of various inflammatory mediators, which induce Ca^2+^ influx by activating the mechanically sensitive TRPV4 channel in SF, accelerating ATP release and ROS production, ultimately enhancing RA synovial cell proliferation (Ref. 83). IL-17A increased TRPV4 expression and neuronal hyperexcitability, and TRPV4 may play an important role in hyperalgesia (Ref. 84). However, a study has shown that TRPV4 activation in the presence of IL-1 reduces IL-8 production, implying that IL-8 production is regulated by TRPV4 under inflammatory conditions (Ref. 85).

Compared with wild-type antigen-induced arthritis (AIA) mice, TRPM2-knockout AIA mice had more obvious knee joint swelling, hyperplasia of knee joint synovial cells and significantly increased synovial tissue inflammatory cell infiltration, IL-6, IL-8, and chemokine (C-X-C motif) ligand 6 mRNA expression levels also increased significantly (Ref. 86). The SF of patients with RA express TRPM3 ion channels. Progesterone sulphate, a TRPM3 agonist, stimulates TRPM3 and inhibits the production/secretion of hyaluronic acid (HA) in RA-FLSs (Ref. 87). HA is secreted by fibroblast-like synovial cells and is a recognized physiological joint lubricant, but excess HA and HA degradation products contribute to RA progression (Ref. 88). TRPM7 is highly expressed in chondrocytes and articular cartilage in an adjuvant arthritis (AA) rat model. Its blockade alleviates chondrocyte apoptosis and articular cartilage damage in AA rats by regulating the Indian Hedgehog signalling pathway (Refs 89, 90). TRPM7 is involved in CD147 (extracellular MMP inducer)-induced chemotaxis, adhesion and invasiveness of neutrophils in patients with RA, leading to progressive cartilage destruction (Ref. 91). In RA synovium, SF are the main cell population of the invasive synovium. TRPM7 expression is significantly enhanced in SF, and TRPM7 channel inhibition can induce SF apoptosis through endoplasmic reticulum stress and exert anti-inflammatory effects (Ref. 92). Menthol-induced SF apoptosis results from TRPM8-mediated extracellular calcium entry, intracellular ROS generation and mitochondrial membrane depolarization (Ref. 93).

TRPA1 activation in sensory nerve fibres causes pain. In a CFA-induced mouse model of chronic arthritis, TRPA1 contributes to joint pain and inflammation (Refs 73, 94, 95). TRPA1 levels are elevated in leukocytes of patients with RA, TNF also upregulates TRPA1 under inflammatory conditions in RA SF, and its activation is accompanied by enhanced calcium influx, decreased proliferation and increased necrosis (Ref. 96).

**Figure 5.**
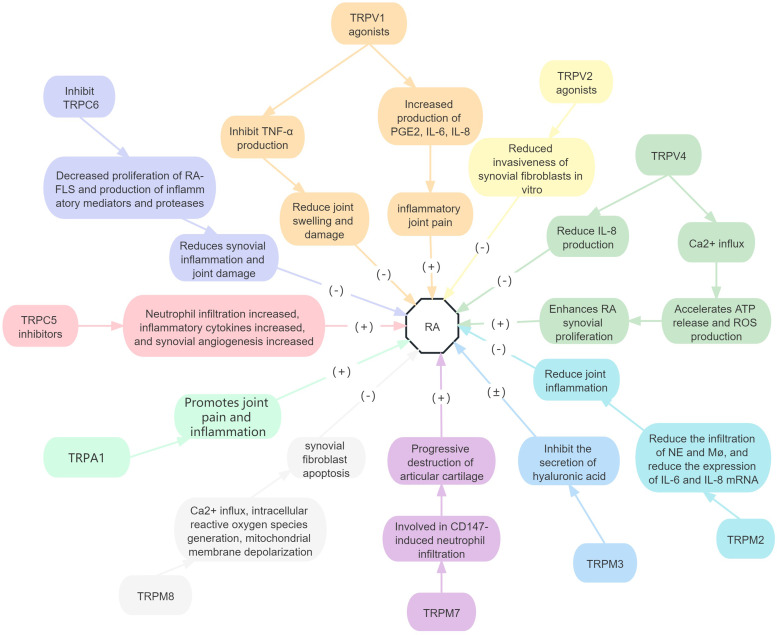
Roles of TRP receptor subtypes in RA. *Note*: (+) means to promote the onset of disease, and (−) means to inhibit the onset of disease. TNF-*α*, tumour necrosis factor alpha; IL, interleukin; PGE2, prostaglandin E2; ROS, reactive oxygen species; NE, neutrophil; Mø, macrophage.

In conclusion, TRPC5, TRPV2, TRPM2 and TRPM8 activation can delay the progression of RA by reducing inflammation and SF invasiveness. However, TRPC6, TRPM7 and TRPA1 promoted RA progression. TRPV1, TRPV4 and TRPM3 have positive and negative effects on RA, which need to be further studied. Other TRP family subtypes have not been identified in patients with RA (Fig. 5).

#### OA

OA is a degenerative disease caused by degeneration of the articular cartilage and reactive hyperplasia of the joint edge and subchondral bone. It often occurs due to ageing, obesity, strain, trauma, joint congenital abnormality, joint deformity and among various other factors. The disease is more common in middle-aged and older adults, as well as in weight-bearing joints and joints with more activities (such as the cervical spine, lumbar spine, knee joint and hip joint). Excessive weight bearing or the use of these joints can promote degenerative changes. Clinical manifestations include a slow developing joint pain, tenderness, stiffness, joint swelling, limited motion and joint deformity. A previous study demonstrated that a variety of immune cells such as macrophages, neutrophils and T cells are involved in OA pathogenesis (Ref. 97).

TRPC5 expression is reduced in the synovial tissue of patients with OA. TRPC5 deletion can promote the release of MMP, a key enzyme for chondrocyte degradation and coordinate immune cell migration and infiltration (Ref. 98).

TRPV1 is expressed in SF from patients with OA. TRPV1 activation can induce the production of inflammatory factors, such as IL-6, thereby helping to regulate the pain sensation in inflamed joints (Ref. 79). TRPV1 expression and M1 macrophage infiltration were simultaneously increased in both human and rat OA synovium. More than 90% of the infiltrated M1 macrophages expressed TRPV1. In the rat OA model, intra-articular injection of capsaicin, a specific TRPV1 agonist, significantly attenuated OA phenotypes, including joint swelling, synovitis, cartilage damage and osteophyte formation. Capsaicin treatment markedly reduced M1 macrophage infiltration in the synovium (Ref. 99). Studies have shown that the infiltration of M1 synovial macrophages and the expression of TRPV4 were increased significantly in OA synovium, inhibition of TRPV4 delays OA progression by inhibiting M1 synovial macrophage polarization through the ROS/NLRP3 pathway (Ref. 100). Meanwhile specific TRPV4 knockout in chondrocytes can reduce OA severity caused by ageing in adult mice and intra-articular administration of a TRPV4 antagonist suppresses pain-related behaviours in a monoiodoacetic acid (MIA)-induced OA pain model (MIA rats), not due to inhibition of knee joint injury or inflammation caused by OA in MIA rats (Refs 101, 102, 103, 104). However, others have shown that TRPV4 agonists can stimulate chondrocytes to produce extracellular matrix, thereby alleviating articular cartilage damage and having a cartilage-protective effect (Refs 105, 106). TRPV5 expression is upregulated in MIA-induced articular cartilage OA. TRPV5 can inhibit cell autophagy by mediating the influx of extracellular Ca^2+^ and increase the production of calmodulin and phosphorylation of calmodulin-dependent protein kinase II (p-CAMK II). Activated p-CAMK II promotes chondrocyte apoptosis through mitogen-activated protein kinases and Akt/mTOR pathways. Previous studies also showed that TRPV5 inhibitors slow down the progression of joint destruction, further demonstrating the role of TRPV5 in OA (Refs 107, 108, 109). In an OA rat model, TRPV6 expression significantly downregulated, resulting in a decreased secretion of extracellular matrix by chondrocytes and a significantly increased expression of MMP-1 and MMP-13, which inhibits chondrocyte proliferation and promotes chondrocyte apoptosis, thereby participating in OA pathogenesis (Ref. 110).

TRPM8 is expressed in human cartilage tissue and on the chondrocyte membrane. Further research has demonstrated that TRPM8 expression in patients with OA was significantly higher than that in healthy people, suggesting that TRPM8 may be related to OA. Cold activation of TRPM8 causes Ca^2+^ overload, increased intracellular ROS production and depolarization of the mitochondrial membrane potential, leading to increased cell necrosis and apoptosis, a potential mechanism (Ref. 111).

TRPA1 mediates sensitization in an OA rodent model (Ref. 112) and induces IL-6 expression in chondrocytes, which in turn upregulates MMP-1 and MMP-13 to promote OA progression (Ref. 113).

**Figure 6.**
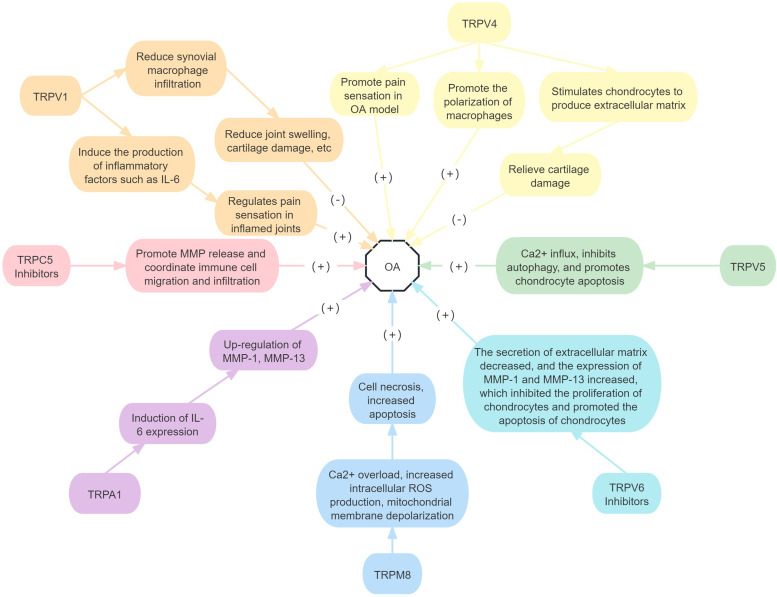
Roles of TRP receptor subtypes in OA. *Note*: (+) means to promote the onset of disease, and (−) means to inhibit the onset of disease. MMP, matrix metalloproteinase; IL, interleukin; ROS, reactive oxygen species.

In summary, the downregulated expression of TRPC5 and TRPV6 in OA may participate in the pathogenesis of OA by promoting MMP release, inhibiting chondrocyte proliferation, and promoting chondrocyte apoptosis. TRPV5, TRPM8 and TRPA1 expression in OA can also contribute to OA pathogenesis by promoting the production of pro-inflammatory factors, and the release of MMP, inhibiting chondrocyte proliferation, and promoting chondrocyte apoptosis. The role of TRPV1and TRPV4 in OA is either positive or negative and requires further study. There are no relevant studies on the roles of other TRP subtypes in OA (Fig. 6).

#### SLE

SLE is an autoimmune inflammatory connective tissue disease that affects multiple organs and occurs mostly in young women. It has a slow onset, insidious occurrence and various clinical manifestations. SLE can involve the skin, serosa, joints, kidneys and central nervous system.

Lupus kidney is the most common visceral lesion in SLE, and the severity of renal lesions directly affects the prognosis. Several studies have shown that TRPC6 gene inhibition can reduce pathological changes such as renal fibrosis (Refs 6, 69, 114, 115) and may delay the progression of lupus kidney. Furthermore, neuropsychiatric lupus erythematosus (NPSLE) is a group of serious complications related to a poor prognosis and high mortality caused by SLE lesions with nervous system involvement, resulting in neurological and/or psychiatric symptoms. Studies have shown that the TRPC6 genotype is associated with NPSLE incidence. Patients with SLE and the TT genotype of the rs7925662 SNP in the TRPC6 gene have an increased risk of developing NPSLE during follow-up, whereas patients with the C allele have a lower NPSLE incidence (Refs 116, 117).

#### SS

SS is a chronic inflammatory autoimmune disease mainly involving the exocrine glands. In addition to impaired function of the salivary and lacrimal glands, resulting in dry mouth and eyes, other exocrine glands and organs outside of the gland are involved, resulting in symptoms of multi-system damage.

In the salivary gland (SG) epithelial cells, IL-17 downregulates TRPC1 expression by inhibiting acetylcholine-induced calcium motility. TRPC1 deletion results in marked attenuation of agonist-induced calcium motility in mice and a 70% loss of salivary secretion (Refs 118, 119). Knockdown of endogenous TRPC1 also significantly reduces store-operated Ca^2+^ entry (SOCE) in the human SG cell lines, mouse pancreatic and submandibular cells. TRPC3 is also involved in SOCE. Mice lacking TRPC1 or TRPC3 show reduced SOCE and glandular secretion, and TRPC3 exerts its function in a TRPC1-dependent manner. TRPC1-knockout mice do not show TRPC3-dependent SOCE (Ref. 120).

TRPV4 activation leads to increased fluid secretion from SG acinar cells by increasing intracellular Ca^2+^. Muscarinic stimulation of salivary and tear secretion was downregulated in TRPV4-deficient mice and acinar cells treated with a TRPV4-specific antagonist (HC-067047). Infusion of the entire submandibular gland with the TRPV1 agonist capsaicin (1 *μ*M) via the submandibular artery significantly increased carbacol-induced salivation, whereas infusion of TRPM8 and TRPA1 agonists decreased it. Moreover, radiation-induced loss of SG fluid secretion is mediated through a TRPM2-dependent pathway that affects mitochondrial function and leads to an irreversible loss of SOCE (Ref. 121).

**Figure 7.**
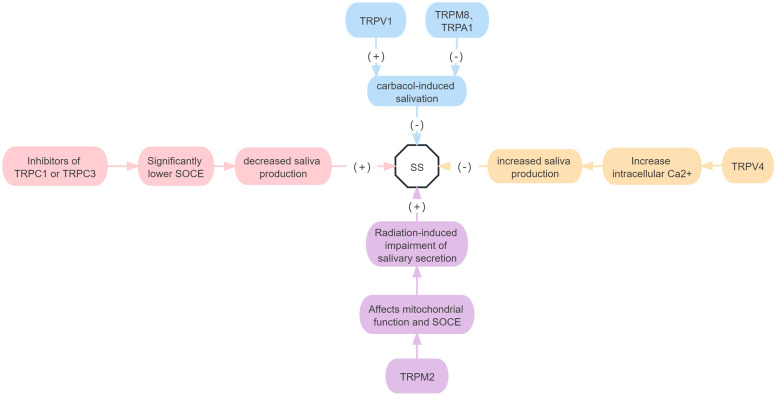
Roles of TRP receptor subtypes in SS. *Note*: (+) means to promote the onset of disease, and (−) means to inhibit the onset of disease. SOCE, store-operated Ca2 + entry.

In conclusion, TRPC1, TRPC3, TRPV1 and TRPV4 all contribute to SG secretion, whereas TRPM2, TRPM8 and TRPA1 inhibit SG secretion. The remaining TRP subfamilies were not related to salivary SG (Fig. 7).

#### Gout

Gout is a common and complex type of arthritis that affects all age groups, with a higher incidence in men than in women. Patients with gout often experience sudden nocturnal joint pain. The onset is acute, and patients experience joint pain, oedema, redness and inflammation. The pain is gradually relieved until it disappears and lasts several days or weeks. Gout is related to an increased concentration of uric acid in the body, and it forms urate deposits in the joint cavity and other locations, thereby causing acute joint pain. Studies have shown that urate deposition in gout can mediate NLRP3 inflammasome activation, leading to the dysfunction of T-cell subsets and the initiation and progression of an autoimmune attack (Ref. 122).

In rats with gouty arthritis, noxious stimulus inducement increases in cerebral blood volume in the primary somatosensory cortex and thalamus. This increase correlates with the upregulation of TRPV1 protein expression and pain behaviour. Selective blockade of central TRPV1 channel activity by intrathecal administration reverses the induced pain and abrogates the cerebral blood volume increase in the thalamocortical region (Refs 123, 124).

Studies have shown that inhibition of TRPM2 channels significantly attenuates monosodium urate (MSU)-induced activation of the NLRP3 inflammasome and macrophages secrete the bioactive substance IL-1*β*. In an animal model of gout, TRPM2 depletion significantly attenuated MSU-induced inflammation dominated by neutrophil infiltration (Ref. 125).

Furthermore, in a mouse model, IL-33 promotes neutrophil migration and triggers neutrophil-dependent ROS production, which in turn activates TRPA1 channels in the dorsal root ganglion neurons and produces pain sensation (Ref. 126). In an additional animal model of MSU-induced gout, MSU injection increased tissue hydrogen peroxide levels, which stimulated TRPA1 and TRPV1 expression on sensory nerve endings, enhanced cellular infiltration and IL-1*β* levels, produced pain sensation and resulted in joint swelling. Both pharmacological inhibition and gene knockout of TRPA1 channels abrogated pain and oedema induced by MSU in animal models (Refs 127, 128, 129).

**Figure 8.**
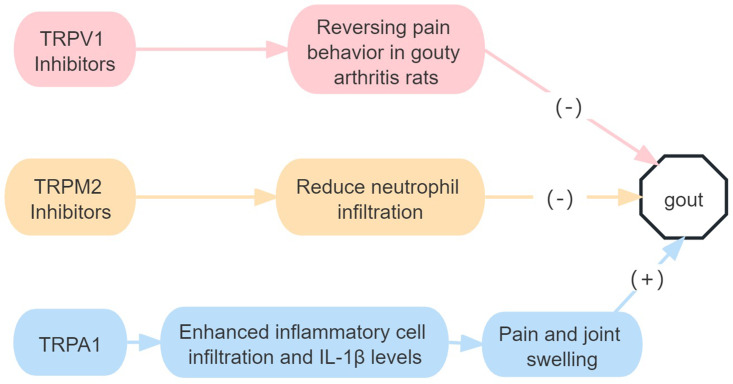
Roles of TRP receptor subtypes in gout. *Note*: (+) means to promote the onset of disease, and (−) means to inhibit the onset of disease. MSU, monosodium urate; IL, interleukin.

In conclusion, TRPV1, TRPM2 and TRPA1 gene knockout or pharmacological inhibition can reduce joint pain and oedema in mice with gouty arthritis. There is no relevant research on the role of the other TRP family subtypes in gout (Fig. 8).

## PIEZO and autoimmune diseases

PIEZO refers to pressure. The PIEZO family has two members, PIEZO1 and PIEZO2. PIEZO1 exists in many cells and tissues of the body, whereas PIEZO2 is mainly related to touch, proprioception, visceral sense and mechanical force perception.

PIEZO1 is widely expressed in the cardiovascular system. It is expressed on vascular endothelial cells, red blood cells, epithelial cells and cardiomyocytes. It also plays an important role in sensing drastic mechanical changes produced by cardiac systolic and diastolic relaxation, thereby regulating cardiac stability. PIEZO1 expression is upregulated in patients with heart disease (Ref. 130). Loss of human PIEZO1 gene function can lead to congenital lymphatic dysplasia, whereas gain-of-function mutations can lead to haemolytic anaemia and hereditary stem cell hyperplasia (Ref. 131). Moreover, PIEZO1 is important for bone formation and remodelling. Long-term bed rest or weightlessness in space can lead to severe bone loss. The reason bones can feel gravity is due to PIEZO1 expressed in osteoblasts. Therefore, PIEZO1 deficiency causes loss of bone mass and spontaneous fractures along with increased bone resorption. Furthermore, PIEZO1-deficient mice have also been shown to develop severe bone loss (Ref. 132).

In contrast, PIEZO2 mediates touch perception and plays an important role in human physiological processes, such as mechanical pain, urination, respiration, blood pressure and bone remodelling. Touching, hugging and the use of various tools, such as the screen of a mobile phone, all require the participation of PIEZO2. There is also ‘proprioception’, our own muscle state, such as maintaining balance when walking, as PIEZO2 in the nervous system can monitor muscle tension (Refs 133, 134). Additionally, mechanical changes in internal organs, such as blood pressure, respiration and bladder filling, are also monitored by PIEZO2 (Refs 135, 136, 137). Loss-of-function mutations in the human PIEZO2 gene cause autosomal recessive muscular dystrophy syndrome with perinatal respiratory distress, joint curvature, scoliosis and possibly urinary abnormalities due to the inability to sense bladder fullness. Gain-of-function mutations, however, cause an autosomal dominant distal joint curvature (Ref. 131).

### Organ-specific autoimmune diseases

Animal studies have shown that the exercise pressor reflex is exaggerated in early T1DM (Refs 138, 139), and PIEZO channels may play an important role in this phenomenon. GsMTx4, a biologically derived peptide that specifically inhibits mechanically activated cation channels, can also inhibit heterologous co-expression of PIEZO1/2, attenuate the exaggerated exercise pressor reflex in T1DM rats and effectively reduce the greater cardiovascular strain caused by intermittent muscle contraction (Ref. 140).

In MS, PIEZO1 channel activation in axons negatively regulates central nervous system myelination (Ref. 141). PIEZO1 depletion in T cells can selectively promote regulatory T-cell proliferation, thereby reducing EAE severity. However, it does not affect thymus development, lymph node homing, T-cell receptor priming, T-cell proliferation and differentiation (Refs 142, 143).

### Systemic autoimmune diseases

A previous study demonstrated that in OA, inflammation enhances mechanical stimulation-induced PIEZO channel currents. However, knee OA does not affect the expression levels of PIEZO1 or PIEZO2 mRNA, suggesting that PIEZO channel function may be enhanced in knee OA without altering the mRNA expression levels (Ref. 144). Though, in OA, IL-1*α* is increased and IL-1*α* inflammatory signalling in articular chondrocytes increases PIEZO1 expression and function in chondrocytes, resulting in increased mechanically induced Ca^2+^ influx, leading to increased sensitivity to mechanical stimuli (Ref. 145). The PIEZO channel mediates the signal transduction of damaging mechanical stimuli. PIEZO1 and PIEZO2 co-expression has a synergistic effect, resulting in increased sensitivity to mechanical stimuli, which in turn promotes chondrocyte injury. However, GsMTx4 attenuates the response of chondrocytes to injury-inducing mechanical stimulation (Refs 146, 147).

## Summary and outlook

The announcement of the 2021 Nobel Prize in Physiology or Medicine brought TRP and PIEZO receptors to our attention. This article summarizes the roles of TRP and PIEZO receptors in various autoimmune diseases. Different subtypes have different roles in autoimmune diseases, some have specific protective effects, and some have harmful effects (Tables 1 and 2). However, most current studies on TRP and PIEZO receptors in autoimmune diseases are animal studies, and there are currently few clinical studies. Therefore, understanding the roles of different receptor subtypes in autoimmune diseases provides a new method for the treatment of autoimmune diseases.

**Table 1.** Role of TRP receptors in organ-specific autoimmune diseases

Disease	Receptor	Mechanism	References
T1DM	TRPC1	The expression of TRPC1 gene in kidney in late DN was significantly decreased, but its genetic polymorphism may not fundamentally promote the development of DN	(Refs 4, 5)
	TRPC6	TRPC6 gene knockout was associated with decreased albuminuria in young animals (12–16 weeks of age) in mouse models, while there was no significant protective effect in rat models	(Refs 6, 7)
	TRPV1	It can significantly reduce islet specific autoimmunity, but stimulate osteoclast generation, which is associated with secondary osteoporosis	(Refs 9, 10)
	TRPM7	Increase fasting insulin levels, reduce blood glucose, ICA, IAA, GAD-Ab levels, improve cognitive dysfunction	(Ref. 11)
T2DM	TRPC1	The CC genotype of rs7638459 significantly increased the risk of T2DM and the CC genotype of rs953239 significantly decreased the risk of DN	(Ref. 12)
	TRPC6	Down-regulation of TRPC6 can improve the apoptosis of renal podocytes in T2DM, reduce calcium overload and neuroinflammation in brain cells and delay the development of cognitive dysfunction associated with T2DM	(Refs 13, 14)
	TRPV1	Antagonists inhibit CGRP secretion, stimulate insulin secretion and reduce insulin resistance	(Refs 15, 16, 17)
		Agonists reduce obesity-induced inflammation, insulin resistance and hepatic steatosis, lower fasting blood glucose levels, and enhance GLP-1 secretion and improve glucose homeostasis	(Refs 18, 19, 20)
	TRPV4	Antagonists reduce high-fat diet-induced obesity, insulin resistance, diabetic nephropathy, retinopathy and neuropathy	(Ref. 21)
	TRPM2	Gene knockout significantly reduced renal fibrosis and inflammation in HFD-induced diabetic mice by inhibiting TGF-*β*1	(Ref. 22)
	TRPM5	Antagonists inhibit insulin release	(Refs 23, 24, 25, 26, 27)
	TRPA1	Insulin resistance can be improved by increasing mitochondrial activity	(Ref. 18)
IBD	TRPC6	Involved in the development of intestinal fibrosis	(Refs 29, 30)
	TRPV1	Gene knockout can alleviate DSS induced colitis and aggravate DNBS induced colitis. The expression of TRPV1 in colonic epithelium of IBD patients is up-regulated. The relationship between TRPV1 expression and disease severity needs further study	(Refs 31, 32, 33, 34, 35, 36, 37, 38, 39)
	TRPV4	The expressions of proinflammatory cytokines and chemokines were up-regulated, and inflammatory cells infiltrated	(Refs 40, 41, 42)
	TRPM2	It plays a pro-inflammatory role through the NF-*κ*B signalling pathway	(Ref. 43)
	TRPM8	The release of TNF-*α*, IL-1, IL-6, MCP-1 and CGRP was inhibited	(Refs 44, 45)
	TRPA1	Colonic neuropeptide release is increased, and its antagonists reduce colitis symptoms	(Ref. 46)
		It can improve neutrophil infiltration in mice induced by DNBS and DSS, reduce the production of cytokines, neuropeptides and chemokines, and has anti-fibrotic effect	(Refs 47, 48, 49, 50)
MS	TRPV1	The NLRP3 inflammatory body activation is regulated by calcium influx and phosphorylation of phosphatase PP2A to participate in EAE and neuroinflammation	(Ref. 52)
		TNF and IL-6 release were significantly reduced	(Ref. 54)
	TRPV2	TRPV2 may play a key role in myelination	(Ref. 55)
	TRPM2	Gene knockout can inhibit the activation of NLRP3 inflammasome, the production of pro-inflammatory cytokines, reduce neutrophil infiltration and delay EAE	(Refs 56, 57)
	TRPM4	Antagonists reduced axonal and neuronal degeneration of EAE and reduced clinical disease scores, but this did not alter EAE-related immune function	(Ref. 58)
	TRPM7	Inhibition of axon growth, kinase mutation can reduce the production of IL-17, IFN-*γ* and other cytokines	(Refs 59, 60)
	TRPA1	Associated with demyelination, PIMS-EAE anxiety, depression and mechanical hypersensitivity to pain	(Refs 61, 62, 64, 65, 66, 67)
GN	TRPC6	Gene knockout alleviated glomerulosclerosis and other pathological changes, but did not reduce total albuminuria, blood urea nitrogen, immunoglobulin and other related indicators	(Refs 6, 69)

**Table 2.** Role of TRP receptors in systemic autoimmune disease

Disease	Receptor	Mechanism	References
RA	TRPC5	Gene knockout can increase the concentration of neutrophil infiltration and pro-inflammatory cytokines, and increase synovial angiogenesis, leading to increased active joint inflammation, hyperalgesia and synovitis	(Ref. 70)
	TRPC6	Inhibition of TRPC6 expression can reduce the proliferation of RA-FLS and the production of inflammatory mediators, and alleviate FLS-mediated synovial inflammation and joint destruction in RA	(Ref. 71)
	TRPV1	Deletion can reduce early joint swelling, synovial inflammation, bone erosion and cartilage damage in CFA-induced joint models, and alleviate later joint pain. Activation of TRPV1 in RA patients promotes the production of prostaglandin E2, IL-6 and IL-8 which mediates pain in inflamed joints	(Refs 72, 73, 74, 75, 79, 80)
		Inhibiting the production of TNF-*α*, reducing the swelling and joint destruction of the hind paw induced by collagen and reducing the production of IL-6, IL-8 and other cytokines and MMP-3 in RA synovium fibroblasts, thus playing an anti-inflammatory role	(Refs 78, 81)
	TRPV2	Reduce invasiveness of synovial fibroblasts in vitro	(Ref. 82)
	TRPV4	Enhanced synovial cell proliferation may play an important role in hyperalgesia, but may reduce IL-8 production under inflammatory conditions	(Refs 83, 84, 85)
	TRPM2	In knockout AIA mice, knee swelling, synovial cell proliferation and inflammatory cell infiltration of synovial tissue were significantly increased. The mRNA expression levels of IL-6, IL-8 and CXCL-6 were significantly increased	(Ref. 86)
	TRPM3	Inhibit hyaluronic acid secretion	(Ref. 87)
	TRPM7	Involved in CD147-induced neutrophil infiltration, which leads to progressive cartilage destruction	(Refs 89, 91, 92)
	TRPM8	Apoptosis of synovial fibroblasts	(Ref. 93)
	TRPA1	It helps with joint pain and inflammation	(Refs 94, 95, 96)
OA	TRPC5	Gene deletion can promote the release of MMP, a key enzyme of chondrocyte degradation and coordinate the migration and infiltration of immune cells	(Ref. 98)
	TRPV1	It can induce the production of inflammatory factors such as IL-6, thus helping to regulate pain perception in the inflammation joint	(Ref. 79)
		The OA phenotype was significantly decreased, and the infiltration of M1 macrophages in synovial membrane was reduced	(Ref. 99)
	TRPV4	Inhibitors inhibit macrophage polarization and reduce joint pain, but do not affect joint damage and inflammation	(Refs 100, 101, 102, 103, 104)
		It can stimulate chondrocytes to produce extracellular matrix, and relieve the injury of articular cartilage, and has the effect of cartilage protection	(Refs 105, 106)
	TRPV5	Inhibit autophagy and promote apoptosis of chondrocytes	(Refs 107, 108, 109)
	TRPV6	Gene downregulation leads to decreased secretion of chondrocyte extracellular matrix, significantly increased expression of MMP-1 and MMP-13, inhibition of chondrocyte proliferation, promotion of chondrocyte apoptosis and further involvement in the pathogenesis of OA	(Ref. 110)
	TRPM8	Ca2 + overload, increased intracellular ROS production and depolarization of mitochondrial membrane potential lead to cell necrosis and increased apoptosis	(Ref. 111)
	TRPA1	The expression of IL-6 is induced, and then MMP-1 and MMP-13 are up-regulated to promote the progression of OA	(Ref. 113)
SLE	TRPC6	It may delay renal lesions, with an increased risk of TT genotype of rs7925662 and a lower incidence of NPSLE in patients with the C allele	(Refs 6, 69, 114, 115, 116, 117)
SS	TRPC1、TRPC3	Involved in SOCE and glandular secretion	(Refs 118, 119, 120)
	TRPV1	Significantly increased salivary secretion induced by carbacol	(Ref. 121)
	TRPV4	Increased intracellular Ca^2+^, resulting in increased fluid secretion in SG acinar cells	
	TRPM2	Involved in radiation-induced impaired salivary gland secretion	
	TRPM8、TRPA1	Reduced the salivary secretion induced by carbacol	
Gout	TRPV1	Alleviating pain	(Ref. 123)
	TRPM2	The activation of NLRP3 inflammasome induced by MSU and the secretion of bioactive substance IL-1*β* by macrophages were reduced, and neutrophil infiltration was alleviated	(Ref. 125)
	TRPA1	Increases inflammatory cell infiltration and IL-1*β* levels, producing pain and causing joint swelling	(Refs 126, 127, 128, 129)
